# Cytokine active disulfide-HMGB1 increased during severe macrophage activation syndrome

**DOI:** 10.1186/1546-0096-12-S1-P57

**Published:** 2014-09-17

**Authors:** Hanna Schierbeck, Erik Sundberg, Karin Palmblad, AnnaCarin Horne, Helena Erlandsson Harris, Jan-Inge Henter, Dan Antoine, Ulf Andersson

**Affiliations:** 1 Women's and Children's Health, Karolinska Institutet, Stockholm, Sweden; 2 Astrid Lindgren's Childrens Hospital, Karolinska University Hospital, Stockholm, Sweden; 3 Medicine, Karolinska Institutet, Stockholm, Sweden; 4 Molecular and Clinical Pharmacology, University of Liverpool, Liverpool, UK; 5 Woman's and Children's Health, Karolinska Institutet, Stockholm, Sweden

## Introduction

Macrophage activation syndrome (MAS) is a life-threatening complication of childhood systemic inflammatory disorders. HMGB1 is a nuclear protein that extracellularly orchestrates key events in inflammation. Recent data revealed that different redox states of three cysteines within HMGB1 render it with mutually exclusive activities: reduced all-thiol-HMGB1 exerts chemotactic activity, disulfide-HMGB1 cytokine-inducing effects, and terminally oxidized sulfonyl-HMGB1 without inflammatory activity.

## Objectives

This study was set to assess the kinetics of HMGB1 in four patients with severe MAS, on the basis of SoJIA (n=3) or SLE (n=1), and to identify which HMGB1 redox isoforms appear during different disease stages.

## Methods

Serial serum samples were analyzed with ELISA for detection of HMGB1, IL-1b, IL-1a, IL-18, IFN-g and MCP-1. Isoforms of HMGB1 were identified and quantified by high-resolution and sensitive proteomic mass spectrometry (MS).

## Results

At onset of MAS three patients had ongoing biologic therapy: two with tocilizumab; one with combination anakinra and CsA. All patients were intensive care treated, three with severe CNS involvement, and all were steroid-resistant. Mass spectrometric characterization revealed early increased levels of predominantly cytokine-inducing disulfide-HMGB1 during severe disease activity. Inflammatory control was achieved in all patients with etoposide, given 50-100mg/m^2^/week. After initiation of treatment and resolving inflammation the HMGB1 levels declined and changed to the non-inflammatory isoform. IFN-g and ferritin appeared concomitant with HMGB1, whereas IL-18 and MCP-1 levels peaked later. As opposed to other studies in MAS, IL-1 could not be detected in any of the patients.

## Conclusion

This work provides new insights in HMGB1 biology suggesting different roles of HMGB1 during the course of the highly inflammatory condition MAS, indicating that the observed elevated HMGB1 levels are not just a product of the inflammation but rather contribute to the development of the cytokine storm seen in MAS patients.

## Disclosure of interest

None declared.

